# Stratifying early‐onset emotional disorders: using genetics to assess persistence in young people of European and South Asian ancestry

**DOI:** 10.1111/jcpp.13862

**Published:** 2023-07-19

**Authors:** Charlotte A. Dennison, Joanna Martin, Amy Shakeshaft, Lucy Riglin, Frances Rice, Cathryn M. Lewis, Michael C. O'Donovan, Anita Thapar

**Affiliations:** ^1^ Wolfson Centre for Young People's Mental Health Cardiff University Cardiff UK; ^2^ Centre for Neuropsychiatric Genetics and Genomics, School of Medicine Cardiff University Cardiff UK; ^3^ Social, Genetic and Developmental Psychiatry Centre, Institute of Psychiatry, Psychology and Neuroscience King's College London London UK

**Keywords:** anxiety, depression, emotional disorder, genetics, prediction

## Abstract

**Background:**

Depression and anxiety are the most common mental health problems in young people. Currently, clinicians are advised to wait before initiating treatment for young people with these disorders as many spontaneously remit. However, others develop recurrent disorder but this subgroup cannot be identified at the outset. We examined whether psychiatric polygenic scores (PGS) could help inform stratification efforts to predict those at higher risk of recurrence.

**Methods:**

Probable emotional disorder was examined in two UK population cohorts using the emotional symptoms subscale of the Strengths and Difficulties Questionnaire (SDQ). Those with emotional disorder at two or more time points between ages 5 and 25 years were classed as ‘recurrent emotional disorder’ (*n* = 1,643) and those with emotional disorder at one time point as having ‘single episode emotional disorder’ (*n* = 1,435, controls *n* = 8,715). We first examined the relationship between psychiatric PGS and emotional disorders in childhood and adolescence. Second, we tested whether psychiatric PGS added to predictor variables of known association with emotional disorder (neurodevelopmental comorbidity, special educational needs, family history of depression and socioeconomic status) when discriminating between single‐episode and recurrent emotional disorder. Analyses were conducted separately in individuals of European and South Asian ancestry.

**Results:**

Probable emotional disorder was associated with higher PGS for major depressive disorder (MDD), anxiety, broad depression, ADHD and autism spectrum disorder (ASD) in those of European ancestry. Higher MDD and broad depression PGS were associated with emotional disorder in people of South Asian ancestry. Recurrent, compared to single‐episode, emotional disorder was associated with ASD and parental psychiatric history. PGS were not associated with episode recurrence, and PGS did not improve discrimination of recurrence when combined with clinical predictors.

**Conclusions:**

Our findings do not support the use of PGS as a tool to assess the likelihood of recurrence in young people experiencing their first episode of emotional disorder.

## Introduction

Genome‐wide association studies (GWAS) of clinical disorders and traits have led to an explosion of research using polygenic scores (PGS; Wray et al., 2021). PGS provide a measure of genetic liability for the disorder or trait in question based on a composite measure of nominally associated common genetic risk variants observed in a discovery GWAS (Wray et al., 2021). There is growing interest in the potential clinical application of PGS across medicine. While most researchers are concerned about the need for caution until PGS are properly evaluated (Kullo et al., 2022), for many disorders, including psychiatric disorders, PGS testing for risk of disorder is already commercially available. One example of a potential early clinical application of PGS relates to coronary artery disease, where risk models appear to be improved by including PGS (Manikpurage et al., 2021; Wray et al., 2021). Risk prediction, in this context, is useful because effective early interventions to reduce risk of myocardial infarction and stroke are available. Subsequently, there is now ongoing evaluation of PGS clinical utility in the context of risk prediction and stratification across many medical disciplines. These are an important next step given concerns about ethical issues and the impact of genetic information, which could be beneficial or harmful (Klarin & Natarajan, 2022; Lebowitz & Ahn, 2017; Murray et al., 2021; Vickers, Calster, & Steyerberg, 2016).

For psychiatric disorders, it seems likely that PGS will have limited utility for predicting onset in the general population (Murray et al., 2021). It is possible that when used in high‐risk samples, PGS could help stratify risk of disorder when combined with clinical variables, contribute to clinical decision‐making, help predict the course of disorder and prognosis and likely effective treatment and risk of side effects (Murray et al., 2021). However, there are many limitations to psychiatric PGS where GWAS have not been as strongly statistically powered as for some physical health disorders, such as coronary artery disease (Wray et al., 2021). Of relevance, PGS are weak predictors of disorder on their own and rely on the discovery and target samples being comparable in terms of ancestry. This is a major problem given that, to date, most GWAS have been conducted on people of European ancestry. Nevertheless, the potential clinical application of PGS, bearing in mind these limitations, needs investigation.

We aimed to examine whether PGS could contribute to stratifying risk of anxiety and depression in young people. We focused on anxiety and depression, collectively emotional disorders, as these are the most common mental health problems in young people and rates have risen sharply in recent years, affecting 18% of individuals aged 7–16 (NHS Digital, 2022; Thapar, Eyre, Patel, & Brent, 2022). For many young people, emotional disorders remit without specialist support or intervention, and accordingly, current guidance to clinicians in the United Kingdom for the management of adolescent depression suggests initially watching and waiting for remission before introducing a psychological intervention (National Institute for Health and Care Excellence, 2019). However, a substantial subgroup of children and adolescents with emotional disorder will have recurrent or persistent illness, which is associated with poorer outcomes (Fergusson, Boden, & Horwood, 2007). At first onset, it is not easy to distinguish between those for whom emotional disorder is a transient or self‐limiting condition of modest clinical significance and those for whom it heralds significant and persistent disorder where intervention is indicated. Clinical studies suggest that a family history of depression, comorbidity and exposure to social adversity are important predictors of persistence or recurrence of depression (Thapar et al., 2022). Recent population and clinic‐based studies also suggest that PGS for both depression and ADHD can, to an extent, predict persistence of depression in young people (Kwong et al., 2019; Rice et al., 2019). This raises the question of whether psychiatric PGS would add to clinical measures for predicting persistence/recurrence of emotional disorders.

In this study, we examine probable emotional disorder in the general population using the Strengths and Difficulties Questionnaire (SDQ; Goodman, 1997). We selected this measure to enable comparison of prevalence rates with the recent Mental Health of Children and Young People Survey (NHS Digital, 2022), which used the same measure, and to enable meta‐analysis across two large, UK population‐based cohorts: the Millennium Cohort Study (MCS) and the Avon Longitudinal Study of Parents and Children (ALSPAC). We first examined whether neurodevelopmental and psychiatric PGS are associated with probable emotional disorder in childhood and adolescence. Second, we tested whether neurodevelopmental/psychiatric PGS added to predictor variables known to be associated with emotional disorder risk (comorbidity, special educational needs, family history of depression and socioeconomic status as a measure of social adversity) when predicting emotional disorder recurrence across childhood and adolescence. ALSPAC also includes data beyond adolescence, so we further investigated persistence up to age 25 years.

## Methods

### Participants

Millennium Cohort Study recruited individuals born between September 2000 and January 2002, who were alive at 9 months and eligible to receive child benefit (Hansen, 2014). At the time of recruitment, almost all children living in the United Kingdom received child benefit, with the exception of a small number of children, such as asylum seekers, who were not eligible (Connelly & Platt, 2014). Children living in economically disadvantaged areas and children from ethnic minority backgrounds were deliberately over‐recruited to better represent typically hard‐to‐reach populations (Connelly & Platt, 2014). At baseline, 18,827 children were recruited, with 10,757 remaining in the cohort by age 17. MCS received ethical approval from the National Health Service Research Ethics Committee at each sweep of data collection. Informed consent was provided by parents/caregivers, and children agreed to participate. From age 16, cohort members provided verbal informed consent to participate in the study. Further details of the ethical approval and consent process can be found here: https://cls.ucl.ac.uk/wp‐content/uploads/2017/07/MCS‐Ethical‐Approval‐and‐Consent‐2019.pdf.

In ALSPAC, pregnant women residing in the Avon area of England with an estimated delivery date between April 1991 and December 1992 were invited to participate in the study. ALSPAC utilised an opportunistic sampling method, placing adverts and recruitment staff in maternity healthcare settings to promote the study. The core sample consisted of 14,541 pregnancies, and of these pregnancies, 13,988 children were alive at 1 year. Following the initial recruitment, an additional 913 children were recruited in three phases resulting in an overall sample of 14,901 children alive at 1 year of age (Boyd et al., 2013; Fraser et al., 2013; Northstone et al., 2019). At age 25, study data were collected and managed using REDCap electronic data capture tools hosted at the University of Bristol. REDCap (Research Electronic Data Capture) is a secure, web‐based software platform designed to support data capture for research studies (Harris et al., 2009).

Ethical approval for the study was obtained from the ALSPAC Ethics and Law Committee and the Local Research Ethics Committees. Informed consent for the use of data collected via questionnaires and clinics was obtained from participants following the recommendations of the ALSPAC Ethics and Law Committee at the time. Consent for biological samples has been collected in accordance with the Human Tissue Act (2004). Please note that the study website contains details of all the data that are available through a fully searchable data dictionary and variable search tool (http://www.bristol.ac.uk/alspac/researchers/our‐data/). Informants include the cohort members themselves, as well as caregivers, siblings and teachers.

### Outcome measures

The SDQ measures mental health difficulties in children and young people, including emotional problems, hyperactivity and conduct problems, as well as prosocial behaviour and peer problems (Goodman, 1997). The emotional symptoms subscale has been validated against diagnoses of anxiety disorders and depressive disorders (Goodman, Ford, Simmons, Gatward, & Meltzer, 2003; Silva, Osório, & Loureiro, 2015). In MCS, the SDQ was collected via parent report at ages 3, 5, 7, 11, 14 and 17 years, and also by self‐report at 17 years. In ALSPAC, the SDQ was collected via parent report at ages 4, 8, 9, 12, 13 and 17 years, and also by self‐report at age 25. Probable emotional disorder was defined as having ever met the ‘high’ cut‐point (a score >4 out of a total of 10) of the emotional subscale of the SDQ, from age 5 to 17 in MCS and age 4 to 25 in ALSPAC. Where self‐report was available, that is, age 17 in MCS and age 25 in ALSPAC, these measurements were used instead of parent report. Individuals who met the threshold twice or more were defined as having recurrent emotional disorder, and those who met the threshold once, at age 14 or younger in MCS and at age 17 or younger in ALSPAC, were defined as having a single emotional disorder episode. We excluded individuals with a first episode during the last sweep of each cohort (i.e. age 17 for MCS and age 25 for ALSPAC) from the recurrence definition, as they had not yet had the possibility to develop a subsequent episode. Thus, the number of individuals included when comparing single‐episode to recurrent emotional disorder will be smaller than the total number of individuals with an emotional disorder.

### Predictor variables

#### Comorbidity

We used the SDQ to define ADHD and conduct problems, in accordance with previous research (Riglin et al., 2021; Stringaris, Lewis, & Maughan, 2014). People were considered to have probable ADHD if they scored >7 for the hyperactivity subscale of the SDQ at least once by the age of 12 – the age symptoms need to onset by to meet DSM‐5 (American Psychiatric Association, 2013) criteria for ADHD. Similarly, individuals who met the cut‐point of >3 on the conduct subscale of the SDQ at least once by the age of 12 were considered to have conduct problems. Autism spectrum disorder (ASD) was defined by parent report of a diagnosis of ASD, at ages 5, 7 and 11 in MCS and age 9 in ALSPAC.

#### Special educational needs (SEND)

In MCS, individuals were classified as having SEND based on parental report at ages 7 and 11 that the school had informed them that the child had SEND. In ALSPAC, individuals were classified as having SEND based on parental report of ever being recognised as having SEND, measured at ages 8 and 11.

#### Parental history of depression or anxiety

Parental psychiatric history was determined by parent report when the cohort members were between the ages of 9 months and 11 years in MCS. Parents were asked whether they had ever been told by a doctor or healthcare professional that they have ‘depression or serious anxiety’. Respondents who were not the biological parent of the cohort member were excluded when defining parental history. In ALSPAC, maternal history of depression or anxiety was defined by parent report between the ages of 12 weeks gestation and when children were 11 years old. Mothers were asked whether they had ever had severe depression, or if they had ever seen a doctor for depression or anxiety.

#### Socioeconomic status (SES)

Socioeconomic status was defined using the Organisation for Economic Co‐Operation and Development (OECD) equivalised income scores at the time of the child's birth in MCS. In ALSPAC, SES was defined on a scale of 1–6 using the mother's social class based on occupation using Office for National Statistics (ONS) guidelines, at the time of the child's birth. SES variables were standardised as *Z*‐scores to allow for comparison between cohorts.

To define each of the above variables, individuals were included if they had data on the relevant item for at least one time point.

### Genetic data

In MCS, saliva samples were provided by cohort members for DNA extraction and genotyping at the age 14 data sweep. Samples were genotyped using Illumina global screening arrays‐24 v1.0 and imputed using the Haplotype Reference Consortium v1.1 panel (Fitzsimons et al., 2021). Imputed genotypes were available for 8,173 cohort members prior to quality control (QC) checks. ALSPAC collected blood samples for DNA extraction either at birth (cord blood) or at study clinics between ages 3 and 7 years. Samples were genotyped using the Illumina HumanHap550 quad platform and imputed using the Haplotype Reference Consortium panel.

Quality control procedures were conducted separately in ALSPAC and MCS, with both cohorts filtered to exclude SNPs on the basis of: INFO <0.8, Hardy–Weinberg equilibrium *p* < 1 × 10^−4^, genotyping rate <0.95 and minor‐allele frequency <0.01. Samples with a call rate of <98% were excluded, alongside duplicate samples and sample mix‐ups identified on the basis of sex and identity‐by‐descent (IBD) checks. Ancestry was determined using the GenoPred pipeline (Pain, 2022), and individuals were assigned to one of five super‐populations (European, South Asian, African, East Asian or admixed‐American). Only individuals of European and South Asian (MCS only) ancestry were retained for further analysis due to insufficient sample sizes for the other ancestry groups. Related individuals were identified using the KING software (Manichaikul et al., 2010), and the individual with the highest genotyping rate from each related family was retained for analysis. Principal components analysis was performed in PLINK v1.9 (Purcell et al., 2007) separately in the European ancestry subset of MCS, the South Asian ancestry subset of MCS, and ALSPAC, using independent SNPs. For further information, see Supplementary Text.

After QC exclusions, 7,142 young people remained in MCS (6,328 European and 814 South Asian ancestry) and 8,648 young people remained in ALSPAC (all European ancestry). PGS were calculated using PRS‐CS (Ge, Chen, Ni, Feng, & Smoller, 2019) based on discovery GWAS for major depressive disorder (MDD; Wray et al., 2018), anxiety (Purves et al., 2020), broad depression (Howard et al., 2018), ADHD (Demontis et al., 2019), ASD (Grove et al., 2019), schizophrenia (Trubetskoy et al., 2022) and bipolar disorder (Mullins et al., 2021). PGS were calculated separately in the European ancestry subset of MCS, the South Asian ancestry subset of MCS, and ALSPAC, and were standardised separately within each of the three groups. Thus, odds ratios should be interpreted as a per‐standard deviation change in PGS. For further information, see Supplementary Text. We calculated scores for both MDD and broad depression due to the differences in case ascertainment across these studies. Wray et al. (2018) included only participants who met a strict diagnostic definition of MDD, while Howard et al. (2018) used a broader definition of depression that encompassed self‐report of depression, as well as ‘anxiety, tension, or nerves’.

### Statistical analysis

#### Primary analysis

Primary analyses were undertaken in individuals of European ancestry in MCS and ALSPAC because of the larger sample size. The two European ancestry cohorts were analysed separately and then meta‐analysed using the R package ‘meta’ (Balduzzi, Rücker, & Schwarzer, 2019) using a fixed‐effects model.

Seven individual logistic regression models were used to test for association between probable emotional disorder and PGS for the seven psychiatric and neurodevelopmental disorders of interest (MDD, anxiety, broad depression, ADHD, ASD, schizophrenia and bipolar disorder), covarying for the first five principal components and any other principal components significantly associated with the outcome. We similarly tested the seven PGS for associations between recurrent and single‐episode emotional disorder using separate logistic regressions for each PGS. We also compared the clinical variables considered: parental history of depression or anxiety, comorbid ADHD, ASD, conduct problems, SEND and SES in those with probable emotional disorder and single‐episode emotional disorder versus recurrent emotional disorder, using a separate logistic regression for each clinical variable. Analyses comparing recurrent to single‐episode emotional disorder were restricted to cases only, with controls (i.e., individuals with no episodes of emotional disorder) excluded.

We used receiver operating characteristic (ROC) curve analyses to compare the power of PGS and clinical predictors to discriminate between recurrent and single‐episode emotional disorder. Individuals without an emotional disorder were not included in the ROC curve analyses. We compared the area under the curve (AUC) of: (a) a model using the PGS with the largest effect on emotional disorder (selected upon analysing PGS against emotional disorder), residualised on the first five PCs, (b) a model using the clinical variables of interest and (c) a combined model including PGS and clinical variables. The MCS European sample was randomly split into a 70% development set and 30% validation set for the ROC curve analyses. The same procedure was applied to ALSPAC to model ROC curves separately in each sample.

Primary results are corrected for multiple testing using false discovery rate (FDR), at a *p*‐value of .05.

#### Secondary analyses

All primary analyses were repeated using individuals of South Asian ancestry from MCS as exploratory analyses owing to the substantially smaller sample size of this subgroup and the absence of a replication sample. Additionally, primary analyses were repeated in a female‐only and male‐only subset of the MCS European ancestry sample to examine potential sex differences in the results.

## Results

The combined sample consisted of 13,752 participants who had at least one rating of the SDQ emotional symptom subscale and passed genomic QC. Key demographics and characteristics are presented in Table 1. In MCS, females were more likely than males to have a probable emotional disorder (odds ratio (OR) = 2.22, 95% confidence intervals (CI) = 2.01–2.46) and recurrent, versus single‐episode, emotional disorder (OR = 2.24, 95% CI = 1.83–2.74). In ALSPAC, females were also more likely than males to have a probable emotional disorder (OR = 2.14, 95% CI = 1.93–2.37) and to have recurrent, compared to single‐episode, emotional disorder (OR = 1.84, 95% CI = 1.50–2.27). In MCS, ancestry was not associated with probable emotional disorder (OR = 0.97, 95% CI = 0.83–1.13), but people of European ancestry were less likely than people of South Asian ancestry to have recurrent, versus single‐episode, emotional disorder (OR = 0.74, 95% CI = 0.57–0.98).

**Table 1:** Number of individuals with available data for each variable who also have PGS available, thus reflecting the number of individuals included in each regression model

Variable	MCS		ALSPAC European ancestry (*n* = 7,616)
	European ancestry (*n* = 5,403)	South Asian ancestry (*n* = 733)	
Sex			
Males	2,649	363	3,851
Females	2,754	370	3,761
Probable emotional disorder			
No emotional disorder	2,798	385	5,532
Emotional disorder	2,605	348	2,084
Recurrent emotional disorder			
Single episode	566	119	750
Recurrent	807	126	710
Parental depression or anxiety			
No parental history of depression or anxiety	2,289	305	5,971
Parental history of depression or anxiety	2,492	222	1,515
ADHD			
No ADHD	4,547	592	6,387
ADHD	851	127	949
ASD			
No ASD	5,230	712	5,741
ASD	152	9	68
Special educational needs			
No SEND	4,645	635	4,021
SEND	695	54	1,387
Conduct problems			
No conduct problems	3,486	431	5,891
Conduct problems	1913	290	1,457
Socioeconomic status	5,211	679	6,316

Socioeconomic status is a continuous measure so the totals refer to number of individuals with any valid score. Total number of individuals with genetic data per sample is displayed at the top of each column.

### Any emotional disorder and PGS

**Figure 1:**
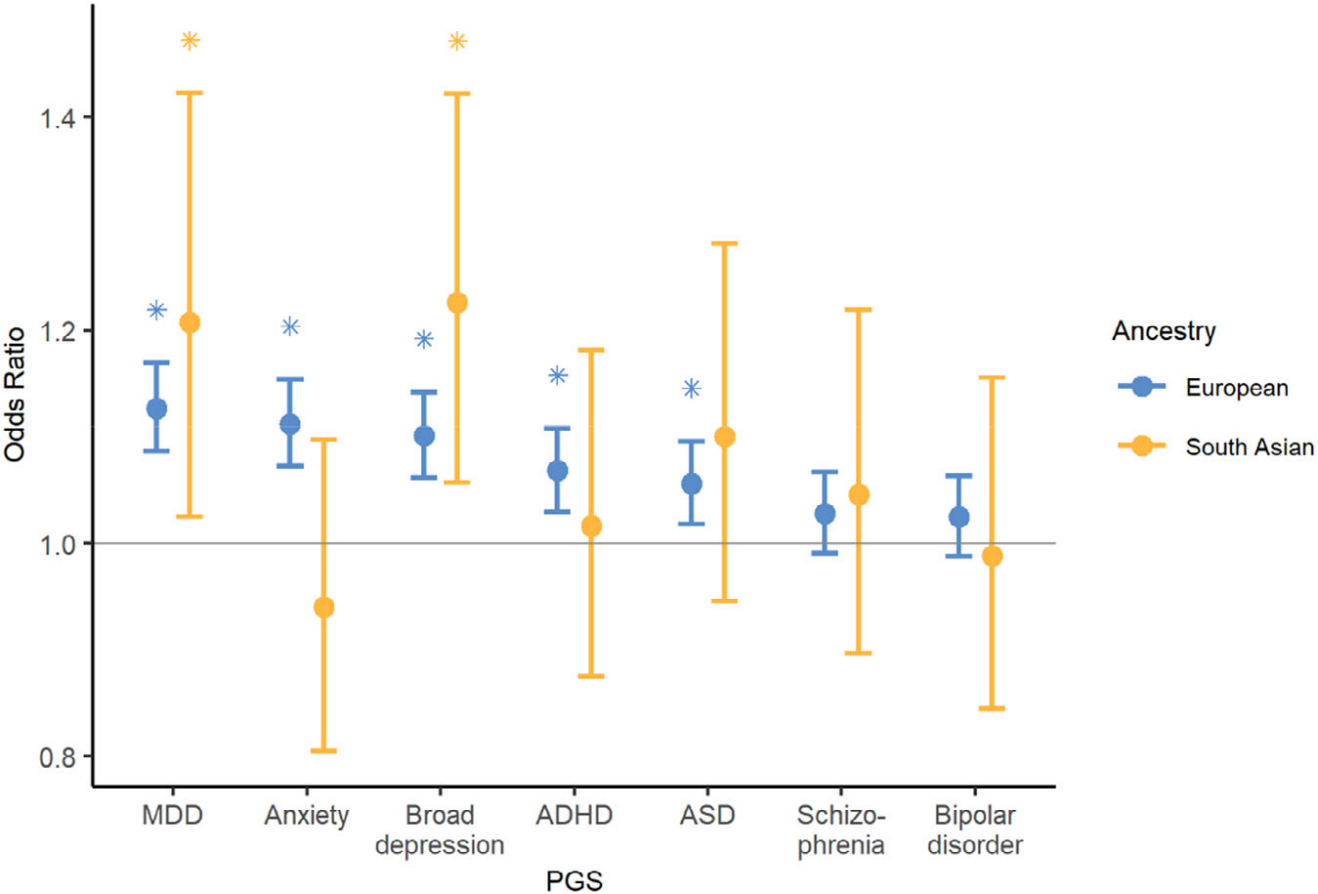
Associations between probable emotional disorder and polygenic scores (PGS) for psychiatric and neurodevelopmental disorders. Blue: European ancestry individuals in MCS and ALSPAC. Yellow: results in South Asian individuals in MCS. Error bars indicate 95% confidence intervals. Statistically significant comparisons are shown with an asterisk [Color figure can be viewed at wileyonlinelibrary.com]

The results of the meta‐analysis of individuals of European ancestry in MCS and ALSPAC and the secondary analysis in individuals of South Asian ancestry are summarised in Figure 1 (see Tables S1–S3 for detailed results).

In the European ancestry samples, probable emotional disorder was associated with higher PGS for MDD (OR = 1.13 [CI = 1.09–1.17], *p* = 1.4 × 10^−9^), anxiety (OR = 1.11 [CI = 1.07–1.15], *p* = 4.9 × 10^−8^), broad depression (OR = 1.10 [CI = 1.06–1.14], *p* = 6.9 × 10^−7^), ADHD (OR = 1.07 [CI = 1.03–1.11], *p* = 7.7 × 10^−4^) and ASD (OR = 1.06 [CI = 1.02–1.10], *p* = 5.2 × 10^−3^). In the South Asian sample, probable emotional disorder was associated with higher PGS for MDD (OR = 1.21 [CI = 1.02–1.42], *p* = 0.02) and broad depression (OR = 1.23 [1.06–1.42], *p* = 0.01; Figure 1 and Table S1).

In the meta‐analysis of European ancestry cohorts, ADHD, ASD, conduct problems, parental psychiatric history and SES were associated with probable emotional disorder (Table S2). In the South Asian ancestry sample, conduct problems were associated with probable emotional disorder (Table S2).

### Recurrent versus single episode of probable emotional disorder

In the European subset of MCS, 1199 individuals had complete data for all clinical predictors and either single‐episode or recurrent emotional disorder (single episode *n* = 483, recurrent *n* = 716). In ALSPAC, 703 individuals had complete data for all clinical predictors, alongside either a single or recurrent episodes of emotional disorder (single episode *n* = 348, recurrent *n* = 355). In the South Asian ancestry subset of MCS, 171 individuals had complete data on all clinical predictors and episode recurrence (single episode *n* = 75, recurrent *n* = 96).

**Figure 2:**
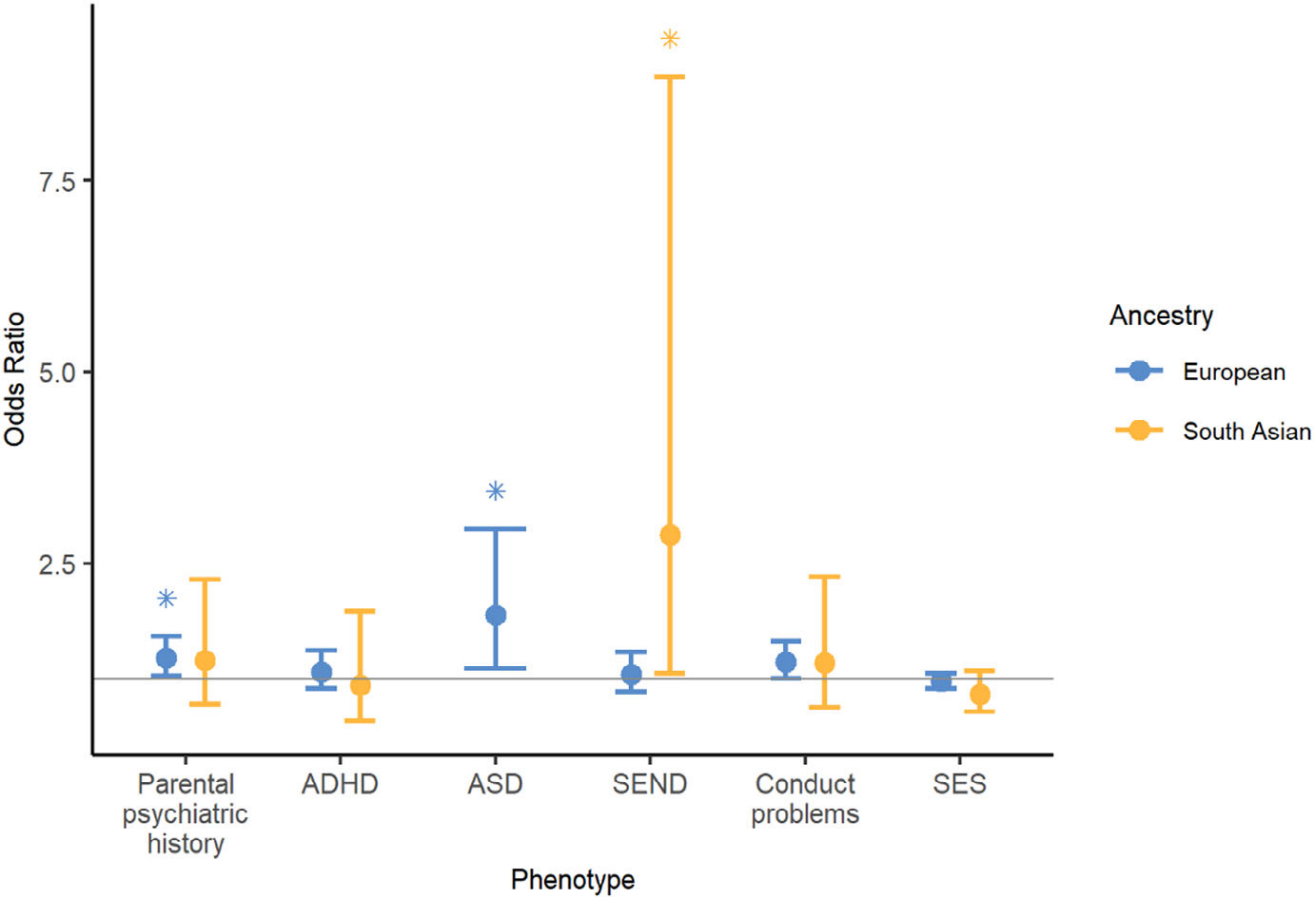
Association between clinical predictors and recurrent, compared to single‐episode, emotional disorder. Error bars indicate 95% confidence intervals. Blue: European ancestry individuals in MCS and ALSPAC. Yellow: results in South Asian individuals in MCS. Statistically significant associations are shown with an asterisk [Color figure can be viewed at wileyonlinelibrary.com]

In the meta‐analysis of individuals of European ancestry, recurrent emotional disorder, compared to single‐episode emotional disorder, was associated with greater likelihood of ASD diagnosis (OR = 1.83 [CI = 1.13–2.95], *p* = .01) and parental history of depression or anxiety (OR = 1.27 [CI = 1.03–1.55], *p* = .02), but not with ADHD, SEND, conduct problems or SES. In individuals of South Asian ancestry, recurrent, compared to single‐episode, emotional disorder was associated with greater likelihood of SEND (OR = 2.88 [CI = 1.07–8.85], *p* = .046), but not with parental history, ADHD, conduct problems or SES (Figure 2 and Table S3).

**Figure 3:**
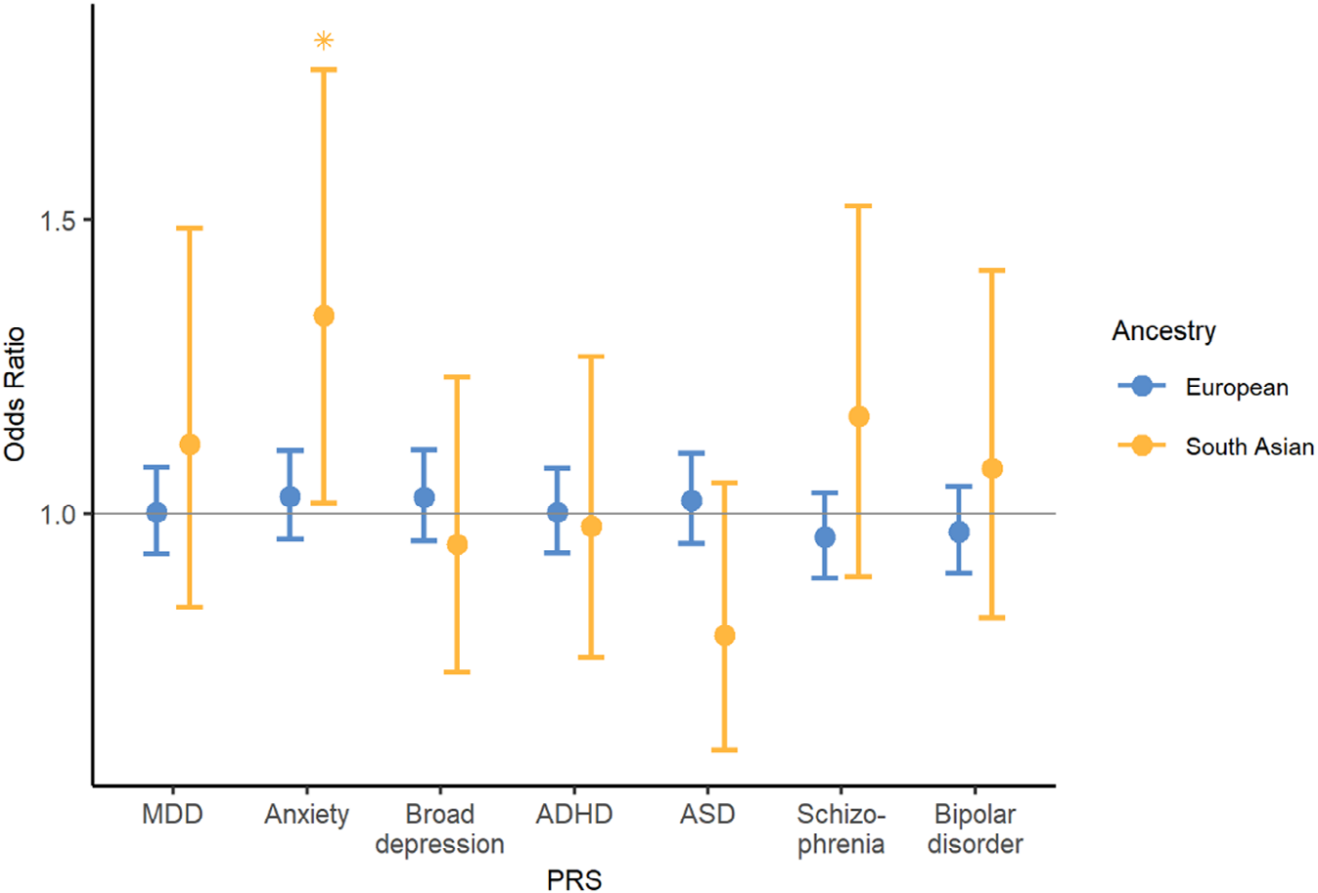
Association between polygenic scores and recurrent, compared to single‐episode, emotional disorder. Error bars indicate 95% confidence intervals. Blue: meta‐analysis between European ancestry individuals in MCS and ALSPAC. Yellow: results in South Asian individuals in MCS. Statistically significant associations are shown with an asterisk [Color figure can be viewed at wileyonlinelibrary.com]

There was no evidence of associations between PGS and recurrent, compared to single‐episode emotional disorder in the European cohort. In people of South Asian ancestry, higher‐anxiety PGS was associated with greater likelihood of recurrent, compared to single‐episode, emotional disorder (OR = 1.34 [1.02–1.76], *p* = .04; Figure 3 and Table S4).

### Combining genetic and clinical data

As MDD PGS showed the largest effect size with any emotional disorder of all the PGS, ROC curve analyses were used to compare the discriminative power of MDD PGS only, clinical predictor variables only and MDD PGS and clinical data combined. In individuals of European ancestry from MCS, the MDD PGS‐only model had an area under the curve (AUC) of 0.53, the clinical data‐only model had an AUC of 0.63 and the combined model had an AUC of 0.64 (Figure S1). In ALSPAC, the MDD PGS‐only model had an AUC of 0.51, the clinical data‐only model had an AUC of 0.58 and the combined model had an AUC of 0.57, demonstrating that including PGS in a model with clinical information did not improve the model's ability to accurately classify individuals as having recurrent or single‐episode emotional disorder.

### Sex differences

The associations between PGS and emotional disorder were consistent between males and females when analysed separately (Tables S5–S7 and Figure S2).

## Discussion

The results showed that PGS for MDD, broad depression, anxiety, ADHD and ASD were associated with having a probable emotional disorder in adolescence and early adulthood, but not with having a recurrent, compared to single‐episode, emotional disorder in people of European ancestry. PGS did not improve prediction of persistence when combined with more readily available clinical predictors, such as neurodevelopmental comorbidities and family history of depression and anxiety, suggesting that, at least for now, PGS are not a useful tool for clinicians in this context.

### Clinical utility of PGS

Despite a substantial amount of research using PGS in psychiatric disorders, clinical applications of PGS have been limited. It has been suggested that PGS may be useful for risk prediction and stratification in psychiatry when used with other information, including family history (Wray et al., 2021). PGS have been associated with depression case–control status in young people (Halldorsdottir et al., 2019), and, when combined with parental psychiatric history and sex, PGS were able to stratify individuals by risk of depression onset before the age of 30 (Agerbo et al., 2021). Consistent with these observations, our results indicate that PGS are also associated with early‐onset emotional disorder. However, PGS were not able to stratify within individuals with an emotional disorder to predict persistence in adolescence and early adult life. In individuals of South Asian ancestry, higher‐anxiety PGS was associated with persistence, however, the wide confidence intervals and small sample size mean that this finding needs to be interpreted with caution. Clinical predictors, including parental history of anxiety and depression and autism, were associated with recurrent emotional disorder, but PGS did not improve the power to discriminate between recurrent and single‐episode emotional disorder when used alongside these measures. Despite observed associations between clinical predictors and persistence, ROC curve analysis could not discriminate between single‐episode and recurrent emotional disorder better than chance. Our findings also indicate that PGS calculated with currently available GWAS summary statistics are not useful for stratifying risk of persistence in children and young people with an emotional disorder, but the presence of family history of depression or anxiety and comorbid neurodevelopmental disorders may indicate greater risk of persistence in this group.

### Neurodevelopmental disorders and recurrent emotional disorder

Research using latent trajectory methods has identified an early‐onset, persistent trajectory of adolescent depression associated with increased ADHD and depression PGS (Kwong et al., 2019; Rice et al., 2019; Weavers et al., 2021). We found probable comorbid ASD to be the strongest predictor of recurrent emotional disorder, and that ADHD and ASD PGS were elevated in individuals of European ancestry with any emotional disorder, compared to control participants. Previous research has suggested a causal effect of ADHD liability on major depression in young adulthood and found that autistic traits differentiate persistence in depression (Riglin et al., 2021; Weavers et al., 2021). Our findings, in conjunction with previous research, indicate that neurodevelopmental disorders may contribute to emotional problems in youth. Further research is needed to determine whether other neurodevelopmental indicators are associated with early‐onset emotional problems, including the role of rare genetic variation in early‐onset emotional disorders.

### Strengths and limitations

A key challenge preventing the clinical translation of genetic findings and exacerbating health disparities is the lack of ancestral diversity in genetic research (Derks, Thorp, & Gerring, 2022). Although we were able to include individuals of South Asian ancestry from MCS in our study, this sample is considerably smaller than the European sample and is underpowered to detect PGS associations. Furthermore, there is a lack of GWAS in South Asian populations, meaning that PGS were generated using summary statistics from predominantly European samples and thus may have a relative lack of power to detect associations in non‐European samples. There is an urgent need for greater ancestral diversity at all stages of genomic research, without which clinical translation and health equality are not possible (Martin et al., 2019).

We chose to define recurrence as the presence of emotional disorder at two or more time points, regardless of the age at which these episodes occurred, for comparability with clinical practice. This may limit our findings as the genetic and environmental factors contributing to recurrence could differ across developmental periods. An alternative approach could be to use latent trajectory methods to identify different developmental patterns of emotional disorders in young people. While a useful approach, trajectories are generated statistically so may be less clinically translatable than using cut points as we elected to do. They also require the same informant across all time points, which may weaken the sensitivity of the SDQ to identify emotional disorder, compared to multiple informants (Goodman et al., 2003). Our findings are, however, similar to those from adolescent depression trajectory‐based research in terms of the observed patterns of associations with psychiatric genetic risk scores (Rice et al., 2019).

Our study primarily used parent‐report measurements to define emotional disorder, with self‐report at ages 17–25. Self‐report measurements were not collected prior to age 17, thus we were unable to compare the validity of parent‐ compared to self‐report. While it is recognised that there are discrepancies between different informants when rating young people's emotional disorder (De Los Reyes & Kazdin, 2004), particularly for younger children, parent ratings are more reliable when compared to gold standard interviews (Lewis et al., 2012), and both parent‐rated and adolescent‐rated emotional disorder predict similar clinical and functional outcomes (Rice, Lifford, Thomas, & Thapar, 2007). Since clinical diagnoses of MDD and anxiety disorders were unavailable in both MCS and ALSPAC samples, we were unable to assess the contribution of PGS and clinical predictors to diagnosed emotional disorders. While the emotional subscale of the SDQ has been validated against diagnoses of anxiety and depressive disorders, it is possible that the broader phenotype used in this study may be less generalisable to clinical populations, compared to diagnosed MDD or anxiety disorders. Furthermore, while our data capture early‐onset emotional disorders, the participants in our study were still in the primary risk period for emotional disorders and some will go on to develop recurrent emotional disorder outside of the time span we have studied. Additionally, investigations of the diagnostic validity of the SDQ have been limited to children and younger adolescents (Goodman et al., 2003), and further investigation of the validity of this measure in individuals aged 16–25 is an important area for future research.

ALSPAC and MCS are both large studies with multiple measures across development, allowing us to study the onset and progression of emotional disorders. While ALSPAC participants are more affluent than the UK population (Boyd et al., 2013), the deliberate oversampling of individuals from typically hard‐to‐reach demographic groups by MCS means the cohort is more representative of the general population (Connelly & Platt, 2014). As longitudinal cohorts, both MCS and ALSPAC are affected by attrition. Although ALSPAC started collecting DNA from birth, MCS did not collect DNA until age 14, and thus the availability of PGS for this cohort will be especially influenced by variables associated with attrition, including elevated schizophrenia PGS (Martin et al., 2016). Similarly, attrition has been associated with emotional disorder (Rice et al., 2019), thus individuals with elevated psychiatric PGS and emotional disorder may be under‐represented in both cohorts. Therefore, it is possible that we underestimated associations among PGS, clinical predictors and emotional disorders, and particularly between PGS and recurrence.

## Conclusions

Advances in the understanding of genetic risks underlying psychiatric disorders, combined with increasing availability of personal genetic testing, mean investigation into the clinical utility of polygenic scores is necessary and timely. Our findings indicate that PGS should not currently be used as a tool to assess the likelihood of early recurrence in young people experiencing their first episode of emotional disorder. Further investigation into potential applications of PGS, as well as prediction models of recurrent depression and anxiety, is needed in both clinical and population cohorts.

## Supporting information


**Table S1.** Emotional disorder at any age predicted by PGS in the meta‐analysed sample as well as separately in each sample/ancestry group.
**Table S2.** Emotional disorder predicted by clinically relevant phenotypes in the meta‐analysed sample as well as separately in each cohort.
**Table S3.** Recurrent emotional disorder, compared to single episode, predicted by clinically relevant phenotypes separately in each cohort.
**Table S4.** Recurrent emotional disorder, compared to single episode, predicted by PGS separately in each Asian cohort.
**Table S5.** Association between PGS and emotional disorder separately in females and males of European ancestry in MCS.
**Table S6.** Recurrent emotional disorder, compared to single episode, predicted by clinically relevant phenotypes separately in males and females of European ancestry in MCS.
**Table S7.** Association between PGS and recurrent emotional disorder separately in females and males of European ancestry in MCS.
**Figure S1.** ROC curves and area under the curve for recurrent emotional disorder.
**Figure S2.** Association.
