# Supplementary material for: Stratifying early‐onset emotional disorders: using genetics to assess persistence in young people of European and South Asian ancestry
Source: J Child Psychol Psychiatry. 2023 Jul 19;65(1):42–51. doi: 10.1111/jcpp.13862 (PMC10807819; doi:10.1111/jcpp.13862)
Supplement: Supplementary file 1 — Table S1. Emotional disorder at any age predicted by PGS in the meta‐analysed sample as well as separately in each sample/ancestry group. Table S2. Emotional disorder predicted by clinically relevant phenotypes in the meta‐analysed sample as well as separately in each cohort. Table S3. Recurrent emotional disorder, compared to single episode, predicted by clinically relevant phenotypes separately in each cohort. Table S4. Recurrent emotional disorder, compared to single episode, predicted by PGS separately in each Asian cohort. Table S5. Association between PGS and emotional disorder separately in females and males of European ancestry in MCS. Table S6. Recurrent emotional disorder, compared to single episode, predicted by clinically relevant phenotypes separately in males and females of European ancestry in MCS. Table S7. Association between PGS and recurrent emotional disorder separately in females and males of European ancestry in MCS. Figure S1. ROC curves and area under the curve for recurrent emotional disorder. Figure S2. Association. [file JCPP-65-42-s001.docx]

**Supporting Information**

Contents

[Supplemental Text 1](#_Toc1288973718)

[Supplementary Tables 4](#_Toc946927481)

[Table S1. 5](#_Toc1726358574)

[Table S2 6](#_Toc1738905435)

[Table S3 6](#_Toc910505740)

[Table S4 7](#_Toc1400626604)

[Table S5 8](#_Toc2104426361)

[Table S6 9](#_Toc186909587)

[Table S7 10](#_Toc497577981)

[Supplementary Figures 11](#_Toc1562211956)

[Figure S1 12](#_Toc600615627)

[Figure S2 12](#_Toc238584702)

# Supplemental Text

*Millennium Cohort Study (MCS) genetic data*

Saliva samples were collected (using Oragene kits) from MCS cohort members (CMs) and their biological parents, at the age 14 data sweep in 2015–2016; for details of data collection and genotyping, see the published report (Fitzsimons et al., 2020). In brief, a total of 21,432 DNA samples from 21,418 individuals were genotyped using the Infinium global screening arrays-24 v1.0 from Illumina. Genotype calling for 21,368 individuals was performed using Genome Studio v2.0.4. Individuals were excluded if they had a missingness proportion >20% or an estimated heterozygosity deviating from the population mean by 5SD (N=207 excluded). Samples were imputed using the Michigan Imputation Server; samples were phased with Eagle.v2.4 (Loh et al., 2016) and imputed to Haplotype Reference Consortium (HRC) release 1.1 (McCarthy et al., 2016) using Minimac.v4 (Fuchsberger et al., 2015).

Following imputation, the imputed dosage data were converted to best guess genotypes (binary format) using PLINK-2 (Purcell et al., 2007a), applying the following quality control (QC) filters: genotype probability <0.9 per individual, missingness>0.02, MAF<0.01, HWE p<10^-4^, and INFO<0.8. Duplicate position and multiallelic SNPs were excluded. Individuals with excessive missingness (>0.05) were excluded (N=97).

Sex chromosomes were not imputed; sex checks based on provided phenotypic sex were performed using the available genotype data for X and Y chromosomes after basic QC (MAF<0.01, missingness>0.01) and LD-pruning (--indep-pairwise 1500 150 0.2). Due to substantial inbreeding in the sample, the F statistic was set higher than the default (--check-sex 0.6 0.8). Samples flagged as showing inconsistent phenotypic and chromosomal sex are suggestive of either a sample mix-up during genotyping or an inaccurately recorded phenotype. All such samples were noted and their relationships were inspected, to determine if a resolution could be reached (see below).

Relatedness analyses were performed using the KING software version 2.2.7 (Manichaikul et al., 2010), to check family relationships by estimating kinship coefficients and inferring identity-by-descent (IBD) segments for all pairwise relationships. For duplicate samples with identical family and person IDs (N=100 pairs), the sample with the higher call rate was kept. For duplicate samples that had different IDs (N=13 pairs), all relationships were inspected and the sample with expected relationships with individuals with the same family ID was kept. All other relationships were inspected and exclusions were made where samples were mixed up and no resolution could be reached (e.g. 2 CMs with an inferred parent-offspring relationship and evidence of sample mix-up based on sex check or no other relationships in the sample); N=13 excluded. Sample IDs or sex were updated if a sample’s identity could be confirmed with certainty (N=60 mixed-up sample IDs were updated and phenotypic sex was updated for 10 confirmed parent samples). Only samples that showed no evidence of sample mix-up and belonged to CMs (based on provided person IDs) or confirmed biological parents of the CMs were retained (N=18,953, including N=8,073 CMs). Mother-father-offspring trios and parent-offspring duo relationships were inferred based on the IBD file to derive sample pedigree information. Mendel analyses in PLINK were used to exclude SNPs with excessive Mendelian errors (>10) and all remaining Mendelian errors were set to missing.

Ancestry of the CMs was identified using the GenoPred pipeline (Pain, 2022) (available at: <https://github.com/opain/GenoPred>) in order to derive homogenous ancestry sub-groups using a reference-standardised approach. Through the pipeline, principal components analysis (PCA) was conducted in a reference sample of known ancestry, 1000 Genomes, on independent SNPs (--indep-pairwise 1000 5 0.2). An elastic net model was used to predict the ancestry of the reference sample using the PCs. PCs were then calculated for CMs and biological parents by projecting PCs from the reference sample onto the target sample and applying these PCs to the elastic net model to predict ancestry. By calculating PCs in the target sample in this manner, individual scores are independent of the other members of the sample and are not affected by relatedness within the sample. The elastic net model calculates a probability of each individual belonging to each ancestral super-population, and assigns each individual to a group based on their most likely group membership. Individuals with <50% probability of being in their assigned population were excluded. Individuals of EAS and AMR ancestry were excluded from genetic analyses due to insufficient sample size. QC was performed on CMs from each of the remaining ancestry subgroups (EUR, SAS, and AFR) using the GenoPred pipeline. K-means clustering was applied to the sample of CMs to define ancestry clusters; outliers were identified and removed based on their distance from the centroid of their assigned cluster.

There were 68 pairs of CMs, comprised of 133 individual CMs, who were related to each other (MZ, DZ/full sibling, half-sibling/cousin). One CM in each related family was retained, with CMs prioritised on the basis of being in a complete trio and by higher call rate, leading to the exclusion of 67 CMs.

The final sample consisted of N=7,325 CMs, which included 3,096 complete parent-offspring trios; see **Table SA** for sample sizes split by ancestry. PCA was re-run using PLINK v1.9 (Purcell et al., 2007b) to obtain ancestry-specific covariates for analyses for each population sub-groups (EUR, AFR or SAS).

**Table SA**

| **Ancestry** | **Cohort members** | | **Trios** |
| --- | --- | --- | --- |
|  | **Males** | **Females** |  |
| European | 3103 | 3082 | 2809 |
| South Asian | 383 | 385 | 254 |
| African | 92 | 83 | 33 |
| Total | 3578 | 3550 | 3096 |

*Polygenic score calculation*

Allelic information was checked against the HRC reference panel and any SNPs with non-matching alleles were excluded. Palindromic or ambiguous (CT/AG) variants were also excluded. Only common SNPs (MAF>0.01) were kept. This resulted in a sample of 3,976,009 SNPs passing all the above QC.

Discovery summary statistics from the largest available published GWAS were used for 7 phenotypes (see **Table SB** for details). The summary statistics were processed to perform QC filtering, align SNPs against the HRC reference panel and convert summary data to a standardised format, using an R pipeline (available at <https://github.com/CardiffMRCPathfinder/summaRygwasqc>).

Polygenic scores (PGS) were calculated for each discovery phenotype in PLINK using the PGS continuous shrinkage (CS) approach (Ge et al., 2019). This is a polygenic scoring approach that uses all available HapMap-3 (Altshuler et al., 2010) SNPs and adjusts the per-SNP effect sizes relative to their GWAS association signals, taking into account linkage disequilibrium (LD), using pre-computed LD information provided with PRS-CS, based on the European ancestry subset of the 1000 genomes phase 3 reference sample. PGS were calculated by summing the number of alleles (weighted by the adjusted effect size) across the full set of SNPs for each person. The PRS-CS-auto approach was used, which automatically detects the sparseness of the genetic architecture for each discovery phenotype, based on the discovery summary statistics. The total number of common autosomal SNPs included in each PGS and the weighted mean value of the shrinkage parameter *phi* (weighted by chromosome size) are shown in **Table SB**.

**Table SB**

| **Discovery phenotype** | **PMID** | **Sample N** | **N SNPs** | **phi** |
| --- | --- | --- | --- | --- |
| Broad depression | 29662059 | 322,580 | 631,824 | 1.3E-04 |
| Anxiety | 31748690 | 114,091 | 562,679 | 1.1E-04 |
| MDD | 29700475 | 173,005 | 619,313 | 1.1E-04 |
| ADHD | 30478444 | 55,374 | 608,646 | 1.2E-04 |
| Autism | 30804558 | 46,350 | 617,442 | 1.1E-04 |
| Schizophrenia | 35396580 | 175,799 | 633,466 | 2.2E-04 |
| Bipolar disorder | 34002096 | 413,466 | 633,073 | 1.5E-04 |

# Supplementary Tables

## Table S1.

| **Group** | **PGS** | **Odds Ratio** | **Lower CI** | **Upper CI** | **P-value** | **FDR P-value** |
| --- | --- | --- | --- | --- | --- | --- |
| Meta-analysis European samples | MDD | 1.13 | 1.09 | 1.17 | 2.0E-10 | 1.4E-09 |
|  | Anxiety | 1.11 | 1.07 | 1.15 | 1.4E-08 | 4.9E-08 |
|  | Broad depression | 1.10 | 1.06 | 1.14 | 2.9E-07 | 6.9E-07 |
|  | ADHD | 1.07 | 1.03 | 1.11 | 4.4E-04 | 7.7E-04 |
|  | ASD | 1.06 | 1.02 | 1.10 | 3.7E-03 | 5.2E-03 |
|  | Schizophrenia | 1.03 | 0.99 | 1.07 | 0.14 | 0.16 |
|  | Bipolar disorder | 1.02 | 0.99 | 1.06 | 0.19 | 0.19 |
| MCS –  South Asian ancestry | MDD | 1.21 | 1.02 | 1.42 | 0.02 | NA |
|  | Anxiety | 0.94 | 0.81 | 1.10 | 0.43 |  |
|  | Broad depression | 1.23 | 1.06 | 1.42 | 0.01 |  |
|  | ADHD | 1.02 | 0.87 | 1.18 | 0.83 |  |
|  | ASD | 1.10 | 0.95 | 1.28 | 0.22 |  |
|  | Schizophrenia | 1.05 | 0.90 | 1.22 | 0.57 |  |
|  | Bipolar disorder | 0.99 | 0.84 | 1.16 | 0.88 |  |
| MCS – European ancestry | MDD | 1.18 | 1.12 | 1.24 | 2.5E-09 | NA |
|  | Anxiety | 1.13 | 1.07 | 1.19 | 7.5E-06 |  |
|  | Broad depression | 1.15 | 1.09 | 1.22 | 2.2E-07 |  |
|  | ADHD | 1.13 | 1.07 | 1.19 | 6.7E-06 |  |
|  | ASD | 1.07 | 1.02 | 1.13 | 0.01 |  |
|  | Schizophrenia | 1.03 | 0.98 | 1.09 | 0.24 |  |
|  | Bipolar disorder | 1.00 | 0.95 | 1.06 | 0.92 |  |
| ALSPAC – European ancestry | MDD | 1.08 | 1.03 | 1.14 | 1.7E-03 | NA |
|  | Anxiety | 1.10 | 1.04 | 1.15 | 3.5E-04 |  |
|  | Broad depression | 1.06 | 1.01 | 1.11 | 3.0E-02 |  |
|  | ADHD | 1.02 | 0.97 | 1.07 | 0.56 |  |
|  | ASD | 1.04 | 0.99 | 1.09 | 0.12 |  |
|  | Schizophrenia | 1.02 | 0.97 | 1.08 | 0.35 |  |
|  | Bipolar disorder | 1.04 | 0.99 | 1.10 | 9.0E-02 |  |

Emotional disorder at any age predicted by PGS, in the meta-analysed sample, as well as separately in each sample/ancestry group. Columns refer to ancestry group, PGS, and odds ratio, 95% confidence intervals, and p-values of the association.

| **Group** | **Phenotype** | **Odds Ratio** | **Lower CI** | **Upper CI** | **P-value** |
| --- | --- | --- | --- | --- | --- |
| Meta-analysis European samples | Parental history of depression or anxiety | 1.57 | 1.42 | 1.73 | 3.5E-18 |
|  | ADHD | 1.56 | 1.35 | 1.79 | 6.3E-10 |
|  | ASD | 2.62 | 1.84 | 3.73 | 9.9E-08 |
|  | Special educational needs | 1.02 | 0.90 | 1.17 | 0.75 |
|  | Conduct problems | 1.45 | 1.30 | 1.61 | 2.5E-11 |
|  | Socioeconomic status | 0.87 | 0.83 | 0.91 | 1.8E-08 |
| MCS –  South Asian ancestry | Parental history of depression or anxiety | 1.28 | 0.88 | 1.85 | 0.20 |
|  | ADHD | 1.20 | 0.72 | 2.00 | 0.48 |
|  | ASD | 1.42 | 0.28 | 10.63 | 0.69 |
|  | Special educational needs | 1.84 | 0.89 | 3.94 | 0.11 |
|  | Conduct problems | 2.15 | 1.46 | 3.19 | 1.1E-04 |
|  | Socioeconomic status | 0.87 | 0.73 | 1.04 | 0.14 |
| MCS – European ancestry | Parental history of depression or anxiety | 1.50 | 1.24 | 1.79 | 1.5E-05 |
|  | ADHD | 1.58 | 1.26 | 1.97 | 6.8E-05 |
|  | ASD | 2.75 | 1.46 | 5.28 | 1.9E-03 |
|  | Special educational needs | 1.00 | 0.83 | 1.20 | 0.98 |
|  | Conduct problems | 1.75 | 1.44 | 2.11 | 9.2E-09 |
|  | Socioeconomic status | 0.92 | 0.85 | 0.99 | 0.03 |
| ALSPAC – European ancestry | Maternal history of depression or anxiety | 1.60 | 1.42 | 1.81 | 3.9E-14 |
|  | ADHD | 1.55 | 1.29 | 1.85 | 2.2E-06 |
|  | ASD | 2.56 | 1.69 | 3.97 | 1.4E-05 |
|  | Special educational needs | 1.04 | 0.86 | 1.26 | 0.66 |
|  | Conduct problems | 1.32 | 1.16 | 1.51 | 3.5E-05 |
|  | Socioeconomic status | 0.84 | 0.78 | 0.89 | 3.4E-08 |

## Table S2

Emotional disorder predicted by clinically-relevant phenotypes, in the meta-analysed sample, as well as separately in each cohort. Columns refer to group, odds ratio, 95% confidence intervals, and p-values of the association.

## Table S3

| **Group** | **Phenotype** | **Odds Ratio** | **Lower CI** | **Upper CI** | **P-value** |
| --- | --- | --- | --- | --- | --- |
| Meta-analysis European samples | Parental history of depression or anxiety | 1.27 | 1.03 | 1.55 | 0.02 |
|  | ADHD | 1.09 | 0.87 | 1.37 | 0.46 |
|  | ASD | 1.83 | 1.13 | 2.95 | 0.01 |
|  | Special educational needs | 1.06 | 0.83 | 1.35 | 0.65 |
|  | Conduct problems | 1.22 | 1.00 | 1.49 | 0.06 |
|  | Socioeconomic status | 0.97 | 0.87 | 1.07 | 0.51 |
| MCS –  South Asian ancestry | Parental history of depression or anxiety | 1.24 | 0.67 | 2.30 | 0.50 |
|  | ADHD | 0.91 | 0.45 | 1.87 | 0.81 |
|  | ASD | Insufficient sample size | | | |
|  | Special educational needs | 2.88 | 1.07 | 8.85 | 0.05 |
|  | Conduct problems | 1.20 | 0.62 | 2.33 | 0.59 |
|  | Socioeconomic status | 0.80 | 0.57 | 1.10 | 0.17 |
| MCS – European ancestry | Parental history of depression or anxiety | 1.33 | 1.03 | 1.72 | 0.03 |
|  | ADHD | 1.08 | 0.82 | 1.43 | 0.58 |
|  | ASD | 1.66 | 0.96 | 2.96 | 0.07 |
|  | Special educational needs | 1.41 | 1.01 | 1.97 | 0.05 |
|  | Conduct problems | 1.18 | 0.92 | 1.52 | 0.18 |
|  | Socioeconomic status | 1.02 | 0.89 | 1.17 | 0.81 |
| ALSPAC – European ancestry | Parental history of depression or anxiety | 1.16 | 0.83 | 1.63 | 0.38 |
|  | ADHD | 1.10 | 0.76 | 1.62 | 0.61 |
|  | ASD | 2.38 | 0.97 | 6.46 | 0.07 |
|  | Special educational needs | 0.77 | 0.54 | 1.10 | 0.15 |
|  | Conduct problems | 1.28 | 0.91 | 1.80 | 0.15 |
|  | Socioeconomic status | 0.91 | 0.78 | 1.06 | 0.21 |

Recurrent emotional disorder, compared to single episode, predicted by clinically-relevant phenotypes, separately in each cohort. Columns refer to group, odds ratio, 95% confidence intervals, and p-values of the association.

## Table S4

| **Group** | **PGS** | **Odds Ratio** | **Lower CI** | **Upper CI** | **P-value** | **FDR P-value** |
| --- | --- | --- | --- | --- | --- | --- |
| Meta-analysis European samples | MDD | 1.00 | 0.93 | 1.08 | 0.97 | 0.97 |
|  | Anxiety | 1.03 | 0.96 | 1.11 | 0.45 | 0.78 |
|  | Broad depression | 1.03 | 0.95 | 1.11 | 0.48 | 0.78 |
|  | ADHD | 1.00 | 0.93 | 1.08 | 0.96 | 0.97 |
|  | ASD | 1.02 | 0.95 | 1.10 | 0.56 | 0.78 |
|  | Schizophrenia | 0.96 | 0.89 | 1.04 | 0.28 | 0.78 |
|  | Bipolar disorder | 0.97 | 0.90 | 1.05 | 0.41 | 0.78 |
| MCS –  South Asian ancestry | MDD | 1.12 | 0.84 | 1.48 | 0.45 | NA |
|  | Anxiety | 1.34 | 1.02 | 1.76 | 0.04 |  |
|  | Broad depression | 0.95 | 0.73 | 1.23 | 0.68 |  |
|  | ADHD | 0.98 | 0.76 | 1.27 | 0.87 |  |
|  | ASD | 0.79 | 0.60 | 1.05 | 0.11 |  |
|  | Schizophrenia | 1.17 | 0.89 | 1.52 | 0.26 |  |
|  | Bipolar disorder | 1.08 | 0.82 | 1.41 | 0.59 |  |
| MCS – European ancestry | MDD | 0.97 | 0.87 | 1.08 | 0.60 | NA |
|  | Anxiety | 1.01 | 0.91 | 1.12 | 0.88 |  |
|  | Broad depression | 1.01 | 0.90 | 1.12 | 0.89 |  |
|  | ADHD | 0.97 | 0.88 | 1.08 | 0.59 |  |
|  | ASD | 1.04 | 0.94 | 1.16 | 0.43 |  |
|  | Schizophrenia | 0.90 | 0.81 | 1.01 | 0.06 |  |
|  | Bipolar disorder | 0.89 | 0.80 | 0.99 | 0.03 |  |
| ALSPAC – European ancestry | MDD | 1.03 | 0.93 | 1.14 | 0.59 | NA |
|  | Anxiety | 1.05 | 0.95 | 1.16 | 0.37 |  |
|  | Broad depression | 1.05 | 0.94 | 1.16 | 0.40 |  |
|  | ADHD | 1.03 | 0.93 | 1.14 | 0.56 |  |
|  | ASD | 1.00 | 0.91 | 1.11 | 0.95 |  |
|  | Schizophrenia | 1.01 | 0.91 | 1.13 | 0.78 |  |
|  | Bipolar disorder | 1.05 | 0.95 | 1.17 | 0.34 |  |

Recurrent emotional disorder, compared to single episode, predicted by PGS separately in each Asian cohorts. Columns refer to ancestry group, PGS, and odds ratio, 95% confidence intervals, and p-value of the association.

## Table S5

| Sex | PGS | Odds Ratio | Lower CI | Upper CI | P-value |
| --- | --- | --- | --- | --- | --- |
| Females | MDD | 1.18 | 1.09 | 1.27 | 2.9E-05 |
|  | ANX | 1.12 | 1.04 | 1.21 | 3.3E-03 |
|  | DEP | 1.14 | 1.06 | 1.24 | 5.4E-04 |
|  | ADHD | 1.16 | 1.08 | 1.26 | 8.9E-05 |
|  | ASD | 1.08 | 1.01 | 1.17 | 0.04 |
|  | SCZ | 1.03 | 0.95 | 1.11 | 0.46 |
|  | BIP | 1.00 | 0.93 | 1.08 | 0.95 |
| Males | MDD | 1.19 | 1.10 | 1.29 | 1.5E-05 |
|  | ANX | 1.15 | 1.07 | 1.25 | 4.0E-04 |
|  | DEP | 1.17 | 1.08 | 1.27 | 8.3E-05 |
|  | ADHD | 1.10 | 1.02 | 1.19 | 0.02 |
|  | ASD | 1.08 | 1.00 | 1.17 | 0.05 |
|  | SCZ | 1.04 | 0.96 | 1.13 | 0.29 |
|  | BIP | 1.02 | 0.94 | 1.10 | 0.70 |

Association between PGS and emotional disorder separately in females and males of European ancestry in MCS. Columns refer to group, the PGS of interest, odds ratio, 95% confidence intervals and p-value of the association.

## Table S6

| Sex | Phenotype | Odds Ratio | Lower CI | Upper CI | P-value |
| --- | --- | --- | --- | --- | --- |
| Females | Parental history of depression or anxiety | 1.47 | 1.03 | 2.10 | 0.03 |
|  | ADHD | 1.75 | 1.11 | 2.80 | 0.02 |
|  | ASD | Insufficient sample size | | | |
|  | Special educational needs | 2.05 | 1.16 | 3.85 | 0.02 |
|  | Conduct problems | 1.13 | 0.80 | 1.59 | 0.49 |
|  | Socioeconomic status | 1.01 | 0.83 | 1.23 | 0.92 |
| Males | Parental history of depression or anxiety | 1.10 | 0.75 | 1.64 | 0.62 |
|  | ADHD | 1.02 | 0.69 | 1.51 | 0.93 |
|  | ASD | 2.18 | 1.20 | 4.05 | 0.01 |
|  | Special educational needs | 1.74 | 1.13 | 2.68 | 0.01 |
|  | Conduct problems | 1.50 | 1.02 | 2.22 | 0.04 |
|  | Socioeconomic status | 1.12 | 0.91 | 1.38 | 0.28 |

Recurrent emotional disorder, compared to single episode, predicted by clinically-relevant phenotypes, separately in males and females of European ancestry in MCS. Columns refer to group, odds ratio, 95% confidence intervals, and p-values of the association.

## Table S7

| Sex | PGS | Odds Ratio | Lower CI | Upper CI | P-value |
| --- | --- | --- | --- | --- | --- |
| Females | MDD | 0.99 | 0.85 | 1.15 | 0.89 |
|  | ANX | 1.03 | 0.88 | 1.19 | 0.73 |
|  | DEP | 1.00 | 0.86 | 1.17 | 0.95 |
|  | ADHD | 0.97 | 0.84 | 1.12 | 0.70 |
|  | ASD | 0.96 | 0.82 | 1.12 | 0.56 |
|  | SCZ | 0.87 | 0.75 | 1.03 | 0.10 |
|  | BIP | 0.84 | 0.72 | 0.98 | 0.03 |
| Males | MDD | 0.93 | 0.79 | 1.11 | 0.43 |
|  | ANX | 1.01 | 0.86 | 1.19 | 0.87 |
|  | DEP | 1.03 | 0.88 | 1.21 | 0.69 |
|  | ADHD | 0.96 | 0.82 | 1.13 | 0.61 |
|  | ASD | 1.16 | 0.99 | 1.36 | 0.07 |
|  | SCZ | 0.93 | 0.79 | 1.09 | 0.34 |
|  | BIP | 0.93 | 0.79 | 1.10 | 0.40 |

Association between PGS and recurrent emotional disorder separately in females and males of European ancestry in MCS. Columns refer to group, the PGS of interest, odds ratio, 95% confidence intervals and p-value of the association.

# Supplementary Figures

## Figure S1


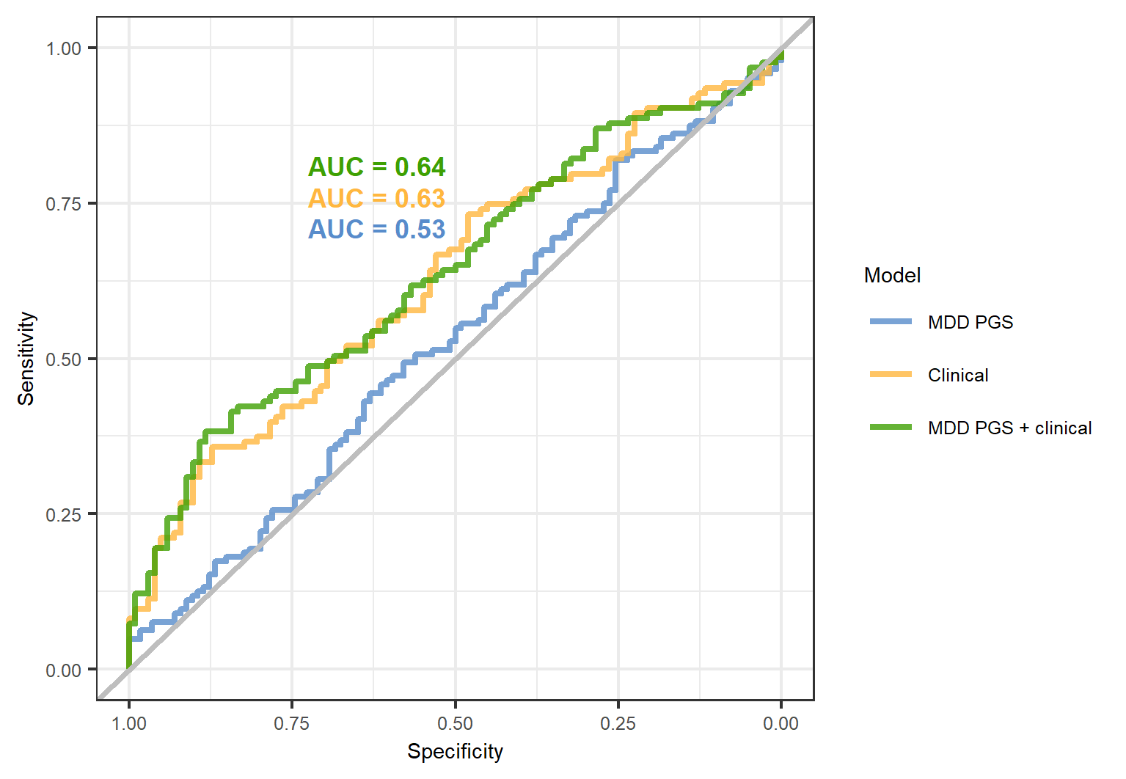
A


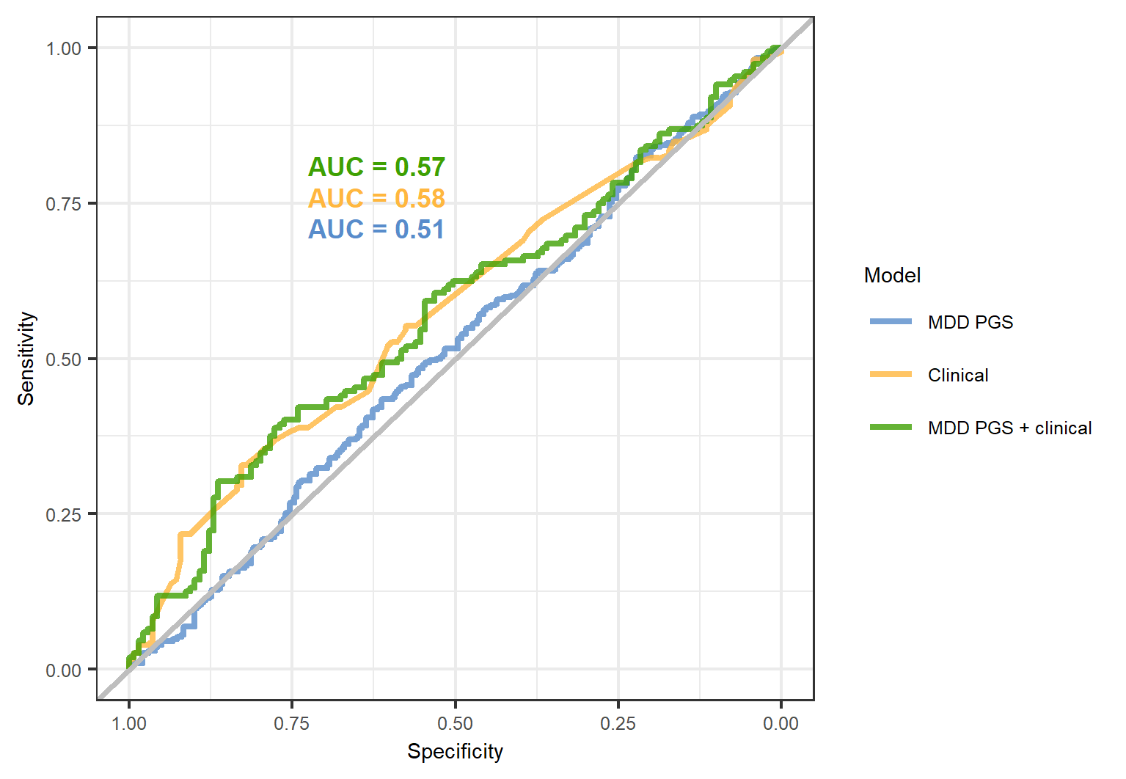
B

ROC Curves and area under the curve for recurrent emotional disorder predicted by: MDD PGS (blue), phenotypes from clinical model (yellow), and MDD PGS with clinical phenotypes (green) in A) MCS and B) ALSPAC.

##
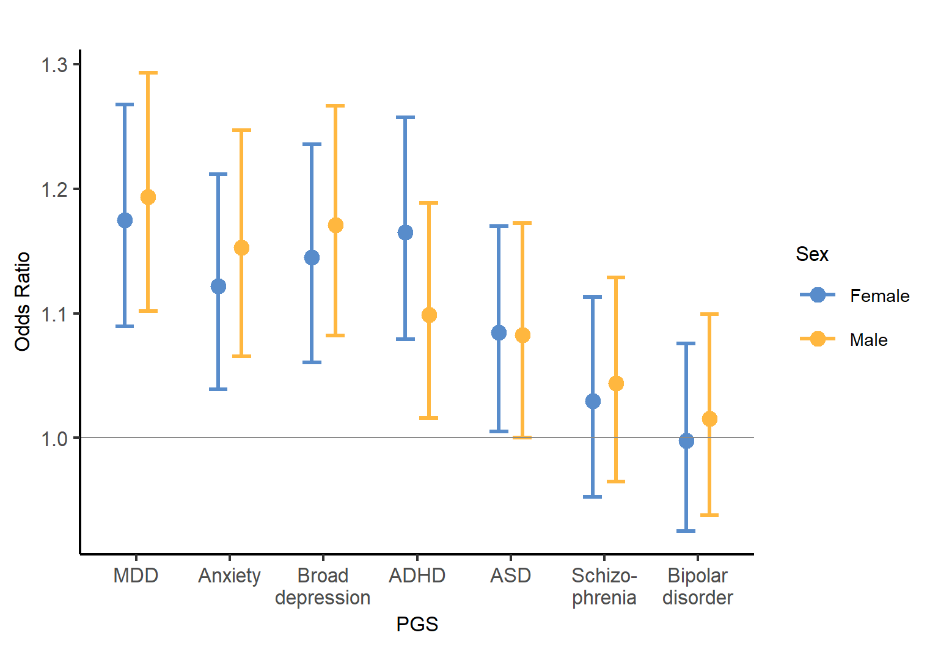
Figure S2

**A**

**B**


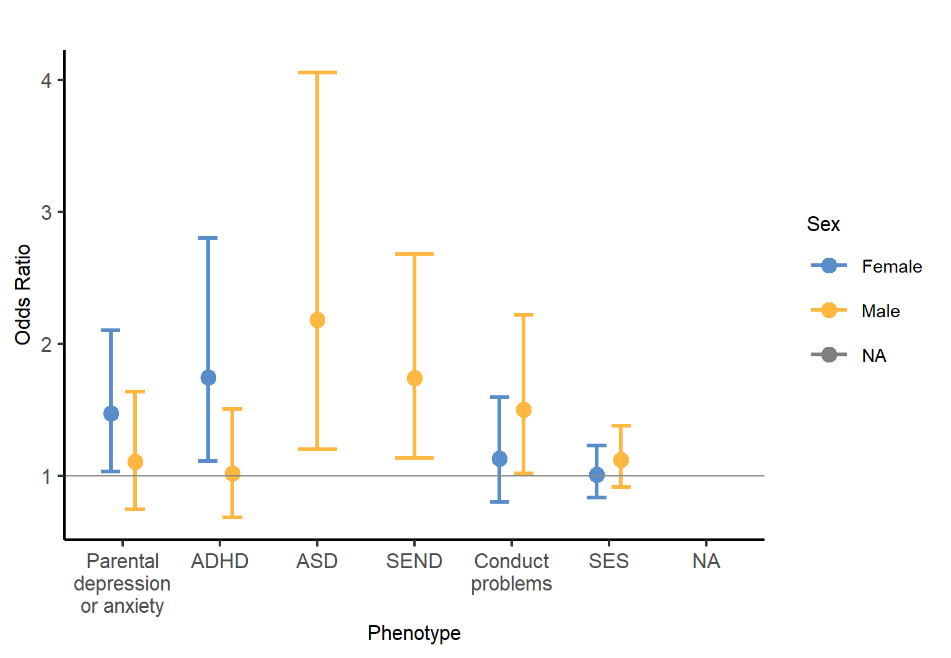


**C**


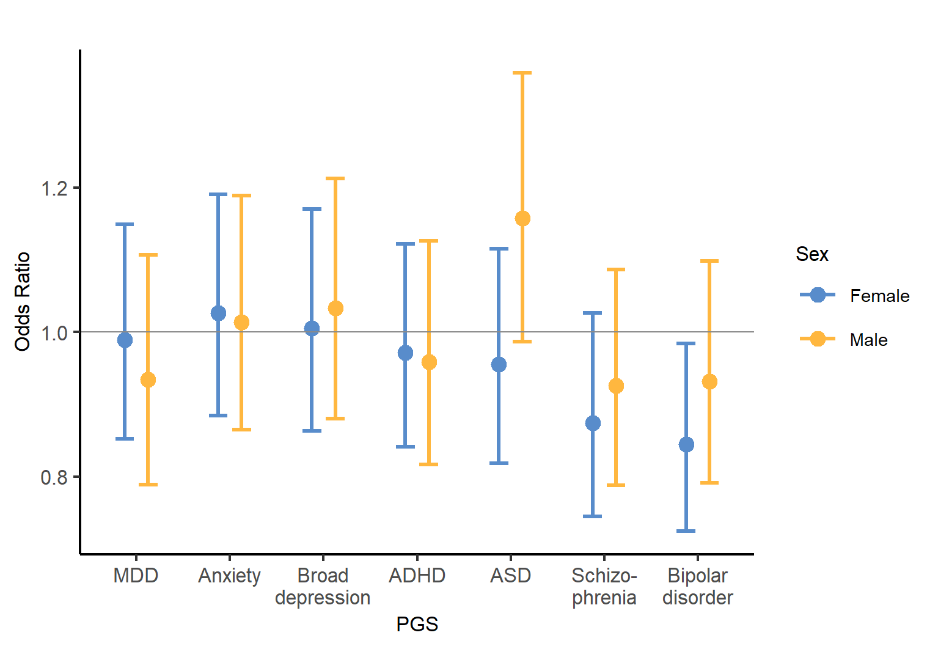


A) Association between PGS and any emotional disorder in males and females. B) Association between clinical phenotypes and recurrent emotional disorder in males and females. C) Association between PGS and recurrent emotional disorder in males and females.
